# An Ikaros Promoter Element with Dual Epigenetic and Transcriptional Activities

**DOI:** 10.1371/journal.pone.0131568

**Published:** 2015-07-02

**Authors:** Elizabeth A. Perotti, Katia Georgopoulos, Toshimi Yoshida

**Affiliations:** Cutaneous Biology Research Center, Massachusetts General Hospital, Harvard Medical School, Charlestown, MA 02129, United States of America; Boston University, UNITED STATES

## Abstract

Ikaros DNA binding factor plays critical roles in lymphocyte development. Changes in Ikaros expression levels during lymphopoiesis are controlled by redundant but also unique regulatory elements of its locus that are critical for this developmental process. We have recently shown that Ikaros binds its own locus in thymocytes *in vivo*. Here, we evaluated the role of an Ikaros binding site within its major lympho-myeloid promoter. We identified an Ikaros/Ets binding site within a promoter sub-region that was highly conserved in mouse and human. Deletion of this binding site increased the percentage of the reporter-expressing mouse lines, indicating that its loss provided a more permissive chromatin environment. However, once transcription was established, the lack of this site decreased transcriptional activity. These findings implicate a dual role for Ikaros/Ets1 binding on *Ikzf1* expression that is exerted at least through its promoter.

## Introduction

A number of stage and lineage specific nuclear regulators govern proper development of blood and immune systems. The Ikaros family of Kruppel-type zinc (Zn)-finger DNA-binding proteins are critical regulators for lymphocyte differentiation and homeostasis [1–3]. They associate with the Nucleosome Remodeling Histone Deacetylace (NuRD) complex in the nucleus [4,5] to affect both gene activation and repression in hematopoietic cells [6–11].

During early hemo-lymphopoiesis, Ikaros is first expressed in the hematopoietic stem cell (HSC) at low levels and then up-regulated in the downstream lymphoid-primed multi-potent progenitor, LMPP [12,13], a major bi-potent progenitor for lymphoid and myeloid lineages [12,14,15]. Up-regulation of Ikaros in the LMPP is critical for priming of the lymphoid program at this stage, and a failure of this event results in a differentiation block towards down-stream lymphoid restricted progenitors such as the common lymphoid progenitor (CLP) and the early thymic precursor (ETP) [12,13,16].

Ikaros is further up-regulated in the quiescent small pre-B cells and double positive thymocytes [1], parallel stages during B and T cell differentiation where lymphoid precursors undergo selection. Reduced Ikaros activity in these precedent stages in differentiation drives malignant transformation of the large-pre-B cells and double negative 3 (pre-T) thymocytes [17–23]; these cells are highly proliferative and are undergoing Rag-mediated recombination of antigen receptor gene loci. Notably, deletions and mutations in the *IKZF1* locus that cause reduction of IKAROS activity are highly associated with development of acute lymphoblastic leukemias in humans [24–28]. Thus, regulatory mechanisms underlying proper expression of Ikaros (IKAROS) at various stages of differentiation are ultimately critical for lymphoid development and homeostasis.

**Fig 1:**
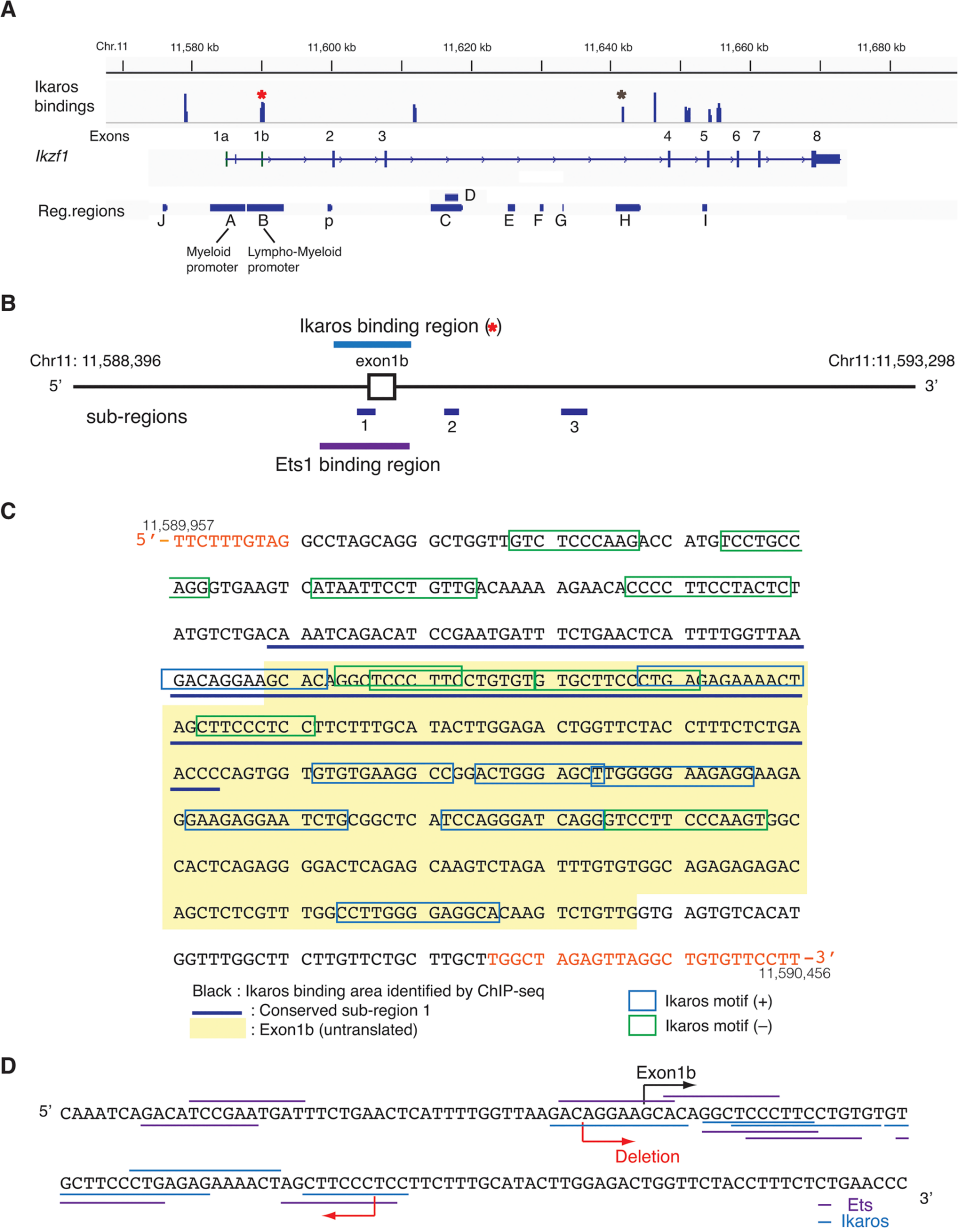
Ikaros binding sites on the *Ikzf1* locus and its promoter region. (A) The The *Ikzf1* locus along with the un-translated exons 1a and 1b[29], and translated exons 2 to 8 [29] are indicated. The known lympho-myeloid specific promoters (A, B), promoter element (p) and enhancers (J, C (D), E, F, H, I) are shown [29,30]. Enrichment for Ikaros bindings on *Ikzf1* revealed by ChIP-seq analysis on wild type thymocytes [11] is indicated (published in [30]). The red and brown asterisks indicate Ikaros binding sites in the promoter-B and enhancer-H regions, respectively. (B) The relative locations of the Ikaros and Ets1 binding regions identified by ChIP-seq [29,30] to the mouse-human homology; sub-regions 1, 2, 3; and exon1b on the promoter-B fragment are shown. (C) The sequence of the Ikaros binding area within the promoter-B region (black letters) is shown. The un-translated exon1b is marked in yellow box, and one of the conserved sub-regions (sub-region 1) is underlined with navy lines. Ikaros binding motifs on the plus strand (+) and minus strand (–) are indicated as blue and green squares, respectively. (D) The sequence of conserved sub-region 1 and the deleted region in the promoter-B of the B-p-GFP parental construct are indicated. Ikaros and Ets consensus binding motifs are underlined by blue and purple, respectively.

In our previous studies, we have established two lympho-myeloid specific promoters, a promoter element, and six enhancers of the *Ikzf1* locus using *in vivo* transgenic reporter mouse systems combined with chromatin studies and comparative genome analyses [29,30] (Fig 1A). Up-stream hemo-lymphoid specific trans-factors that bind the *Ikzf1* locus *in vivo* have been identified in hematopoietic lineages [30], however, the actual roles of these factors on *Ikzf1* regulation remain unknown. One of these factors is Ikaros itself, suggesting an auto-regulatory function of the locus [30]. Another factor is Ets1 that is implicated in various immune functions [30,31]. Ets1 shares its consensus binding motifs with those of Ikaros [32].

Here, we explored the roles of Ikaros in its own expression. An Ikaros/Ets1 binding site was located in the major promoter region and subjected to examination for its role in transgenic reporter expression. Our results implicate that Ikaros and/or Ets1 play a role in both repression and activation of *Ikzf1* expression through its promoter element.

## Materials and Methods

### Mice

The animal protocol was approved by the Subcommittee on Research Animal Care (SAC) of the Massachusetts General Hospital (MGH), which serves as the Institutional Animal Care and Use Committee (IACUC). The transgenic GFP reporter lines (C57BL/6×C3H) B-p-GFP[29] and BΔIK-p-GFP were bred and maintained under specific pathogen free conditions in the animal facility at the MGH, Bldg. 149 in Charlestown, MA. The mice were 4–12 weeks of age at the time of analysis. Euthanasia of animals under this study was performed by CO_2_ asphyxiation. This method was consistent with the American Veterinary Medical Association (AVMA) panel on euthanasia and was simple and humane.

### ChIP-sequencing

ChIP-sequencing data was obtained from Zhang et al. (2011) [11] and ENCODE project [33] (published in [30]). The data were visualized and analyzed using IGV genome browser (Broad Institute) using the mouse genome assembly mm9.

### Transcription factor binding motif search

Putative transcription factor binding motif search was performed using TRANSFAC and RSAT programs (http://www.rsat.eu).

### Generation of the BΔIK-p-GFP reporter line

A cluster of five Ikaros binding motifs in the conserved sub-region 1 was deleted using Quick-change site-directed mutagenesis XL kit (Stratagene) from B-p-GFP construct[29]. The primer sequences used are: R10delta4IkF; 5’- TTCTGAACTCATTTTGGTTAAGACACCTTCTTTGCATACTTGGAGACTGG-3’ and R10delta4IkR; 5’-CCAGTCTCCAAGTATGCAAAGAAGGTGTCTTAACCAAAATGAGTTCAGAA- 3’. The transgene was released from pBluescript II KS (+) vector backbone and purified for microinjection. Transgene positive founder lines were identified by genomic PCR using a primer set to detect EGFP as previously described [29].

### Flow Cytometric analysis

Flow cytometry was performed using fluorescent antibodies from BD Bioscences, Caltag or eBiosciences specific to the following surface antigens: B220 (RA3-6B2), CD4 (RM4-5), CD8α (53–6.7), TCRβ (H57-597), Mac-1 (M1/70), Gr-1 (RB6-8C5). Antibody-stained cells were analyzed using FACScan, FACS Calibur, FACSCanto flow cytometers (BD). Data analysis was performed using FlowJo software (Tree Star, Inc.).

### Statistical analysis

The statistic significance for GFP-expression between the B-p-GFP lines and BΔIK-p-GFP lines was tested using chi-square analysis that was performed using software at (http://www.physics.csbsju.edu/stats/contingency_NROW_NCOLUMN_form.html). Significance was determined if p< 0.05.

## Results

### Analysis of Ikaros binding sites at the *Ikzf1* locus

Murine Ikaros is encoded at the *Ikzf1* locus on chromosome 11. Previous studies have identified two hematopoietic tissue specific promoters (A, B) in un-translated exons 1a and 1b regions, a promoter element (p) just up-stream of the first translated exon 2, seven translated exons (2 to 8), and six lympho-myeloid specific enhancers (J, C (D), E, F, H, I) on the locus (Fig 1A) [29,30]. Our chromatin immunoprecipitation sequencing (ChIP-seq) studies using thymocytes have mapped eight Ikaros binding sites at the *Ikzf1* locus [30], suggesting the possibility of auto-regulation (Fig 1A). One of these binding sites resides within one of the *Ikzf1* promoter regions active in lymphoid and myeloid cells (promoter-B) [29]. Another binding site is within the enhancer-H region active in T cells [30] (Fig 1A). The remaining binding sites did not show overlap with known regulatory regions of *Ikzf1*.

Additional analyses of these binding sites revealed that the Ikaros binding site in the promoter-B region (Chr.11: 11,589,967 to 11,590,431) spanned the un-translated exon 1b and overlapped with one of the mouse-human conserved sub-regions (sub-region 1; Chr.11: 11,590,065 to 11,590,211) in the promoter-B region (Fig 1B and 1C). In contrast, the binding site in the enhancer-H (Chr.11: 11,641,742 to 11,641,921) did not show overlap with any of the mouse-human conserved sub-regions of the enhancer-H (sub-region 1: Chr.11: 11,640,927 to 11,641,042, sub-region 2: Chr.11: 11,642,903 to 11,642,951, sub-region 3: Chr.11: 11,644,262 to 11,644,302). Inspection for transcription factor binding motifs within the Ikaros-binding site in the promoter-B region identified a number of Ikaros binding consensus motifs, consistent with the previously described binding mode of Ikaros to tandem sites as oligomers [4,11,34,35] (Fig 1C).

### A dual function of the Ikaros/Ets1 binding site for reporter expression through promoter-B region

Cross-species conserved regions often contain regulatory elements. Thus, the Ikaros binding site in the promoter-B region overlapping with the conserved sub-region 1 was further examined for its role in auto-regulation *in vivo*.

**Fig 2:**
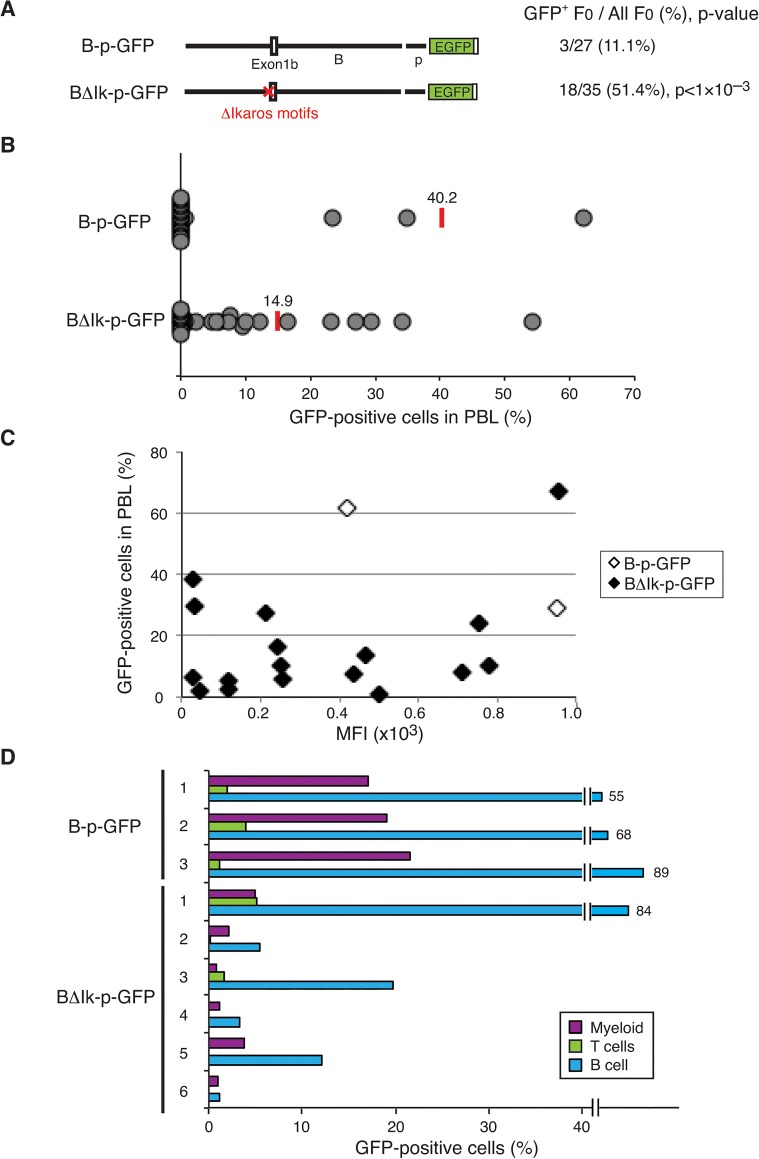
Deletion strategy to generate BΔIK-p-GFP construct and activities of the BΔIK-p-GFP transgene in mice. (A) Diagrams of the parental B-p-GFP [29] and BΔIK-p-GFP constructs are depicted on the left. The number of GFP-expressing (>1% of PBL) founders is displayed over the total number of transgene positive founders obtained for each reporter construct. The statistical significance of the increase observed with BΔIK-p-GFP compared to the parental B-p-GFP was provided by chi-square analysis and is shown as a p-value. (B) The percentage of GFP^+^ PBL assessed by flow cytometry is depicted for each founder line as grey circles. The average percentage of GFP^+^ PBL for each transgenic reporter line was calculated for all the GFP-expressing founders and shown as the red bars and numbers above. (C) The median fluorescent intensity (MFI) of GFP^+^ PBL from GFP-expressing animal is plotted against the percentage of GFP^+^ PBL. White diamonds, parental B-p-GFP founder lines; black diamonds, BΔIK-p-GFP founder lines. The MFI for one of the parental lines was not available at the time of the analysis. (D) Cell-type specific GFP reporter expression in the PBL in six BΔIK-p-GFP lines compared to the three parental B-p-GFP lines. B cells, T cells and myeloid cells were detected as B220^+^, TCRβ^+^ and Mac-1^+^ cells within PBL, respectively.

Six Ikaros consensus binding motifs were deleted from the promoter region in the context of the B-p-GFP cassette and transgenic mice were generated (Fig 1D and 2A left, BΔIK-p-GFP). Ikaros consensus binding motifs are often identified as Ets consensus binding motifs, and these deleted motifs in the promoter region largely overlapped with Ets motifs (Fig 1D). The investigation for Ets1 occupancy on *Ikzf1* has shown that this promoter region is also enriched for an Ets1 binding in a B cell line (Chr.11: aprox.11,589,800 to 11,590,500) (Fig 1B) [30]. Thus, the deletion of this site (referred as Ikaros/Ets1 binding site hereafter) might not only affect Ikaros binding but also Ets1 binding in reporter gene expression.

The parental promoter-only construct B-p-GFP was highly susceptible to the transcriptionally restrictive chromatin environment and only three out of twenty-seven transgene-positive mice showed more than 1% of GFP expression in peripheral blood leukocytes (PBL) that contain B cells, T cells and myeloid cells as previously described [30]. In contrast, of thirty-five transgene positive lines obtained from BΔIK-p-GFP construct, eighteen lines showed more than 1% of GFP expression in PBL (Fig 2A). This was a significantly higher proportion compared to the parental B-p-GFP reporter (51.4% vs 11.1%, *p*<10^−3^).

Nonetheless, expression in PBL was variegated to an even greater level compared to the expressing founders generated by the parental reporter despite the greater number of expressing founders generated by the BΔIK-p-GFP, which was shown by a lower average GFP expression among expressing lines (Fig 2B, 14.9% vs 40.2%).

This indicates that in the context of the *Ikzf1* promoter, the Ikaros/Ets1 binding site may play two roles with respect to transcriptional outcome. While it may promote the recruitment of negatively acting chromatin factors and a transcriptionally restrictive chromatin environment, if this activity is overturned and once a permissive chromatin is established, it may promote recruitment of positive acting transcription regulators.

The median fluorescent intensity (MFI) of reporter expression, which is a measure for transcription rate, was evaluated for GFP-expressing founder lines. Two out of three parental B-p-GFP lines were available for this analysis with eighteen BΔIK-p-GFP lines (Fig 2C). Although the majority of the BΔIK-p-GFP lines seemed to show lower MFI than the parental lines, the number of the parental lines was too small to show any statistical significance. When the MFI of a parental line vs. BΔIK-p-GFP lines with a similar frequency of GFP expression were compared, there was no trend in two different comparisons; in one case (~30% in GFP expression), the MFI of the parental line was higher than three BΔIK-p-GFP lines while in another case (~60% in GFP expression), the MFI of the parental line was lower than the BΔIK-p-GFP line. The MFI vs. frequencies of GFP^+^ cells among all the founder lines also did not show any correlation.

### No T cell specific effect of the Ikaros/Ets1 binding site in the promoter-B region

The promoter B is active from the HSC and progenitors to B cells, myeloid cells, and pre-T cells; however, it does not support reporter expression past pre-T cell stages without the T cell specific enhancers-D or-H. Therefore, the parental B-p-GFP shows the expression profile; “high B/low T/intermediate myeloid” in PBL in all three lines [29,30] (Fig 2D). These results prompted further investigation of cell type specific repressive activities of the Ikaros/Ets1 binding site in the promoter-B.

In the six GFP-expressing BΔIK-p-GFP lines tested, four lines maintained the expression profile of the parental B-p-GFP reporter line “high B/low T/intermediate myeloid” and two lines showed GFP expression predominantly in B cells (Fig 2D). This indicated that the Ikaros/Ets1 binding site tested was not likely providing the cell type specific repressive effects on *Ikzf1* expression past pre-T cell stages. This site is also likely to provide more stable reporter expression in B cells and myeloid lineages as seen in overall decrease in the percentage of GFP^+^ cells in each cell type of the BΔIK-p-GFP lines tested.

## Discussion

Changes in Ikaros expression correlate with its critical roles at various stages of lymphopoiesis. Characterizing cis-regulatory elements on *Ikzf1* provides a better understanding on molecular mechanisms that control proper expression of *Ikzf1* during lymphocyte development. Here, we investigated the function of the Ikaros/Ets1 binding site in *Ikzf1* promoter region using transgenic fluorescent reporter mice. We have provided evidence that the Ikaros/Ets1 binding site tested have a dual role in controlling reporter expression. Given Ikaros and Ets1 are both expressed in B lineages and T lineages from progenitor stages (http://www.immgen.org) [36], current results suggest Ikaros and/or Ets1 regulate *Ikzf1* expression at least through its promoter region. Elucidating Ikaros and Ets1 occupancies in different lymphoid cell types remain elusive to understand their stage specific roles.

Ikaros/Ets1 on one hand may recruit factors promoting restrictive chromatin. Given the parental promoter-only B-p-GFP construct was highly susceptible to a repressive chromatin environment, it revealed de-repression of the GFP reporter upon the deletion of the Ikaros/Ets1 binding site resulting in a greater number of GFP-expressing lines. The parental transgene may be expressed only when the negative effect of Ikaros and/or Ets1 is overcome in a permissive chromatin environment.

In contrast, Ikaros/Ets1 may recruit factors to positively regulate transcription once the locus becomes accessible. Since transcription is regulated in a stochastic manner at the single cell level [37], Ikaros/Ets1 may increase the probability of reporter expression in a permissive environment. This was shown by the increased varigation upon the deletion of the binding site.

Due to the limited number of available GFP-expressing parental lines, it was not conclusive whether the lack of the site affected transcription rate of reporter expression. Nonetheless, there was no trend between the MFI and frequencies of the GFP^+^ cells among all the mice, or when similarly expressed parental and mutant lines were compared. Therefore, probability of expression and transcription rate may be independently regulated, and the Ikaros/Ets1 binding in the promoter may not affect transcription rate.

The repressive effect of the Ikaros/Ets1 binding site is likely to be counteracted by activities of stage and cell type specific cis-elements and trans-factors. However, inactivation of the reporter in mature T cells was not due to the repressive effect; rather *Ikzf1* expression in T-lineages requires additional positive regulation by enhancers-D or-H.

In the HSC, Ikaros may play a negative role in order to maintain its expression low in this stage. In order to overcome the repressive or poised state and move into a downstream lymphoid pathway, additional factors such as E2A, a critical regulator for the proper differentiation of the LMPP [38], may be required for up-regulation of Ikaros. Since E2A consensus motifs were frequently found in adjacent to Ikaros’ motifs [38], these two factors may cooperate together for *Ikzf1* regulation. Once Ikaros expression is established in lymphoid progenitors, Ikaros may play a positive role to further up-regulate its expression at lymphoid precursor stages

Further investigation on the roles of cis-elements and trans-factors including E2A, Ets1 and Ikaros will provide insights on transcriptional networks underlining *Ikzf1* expression during lymphocyte differentiation. This will aid manipulation of Ikaros expression levels in normal and malignant lymphoid lineages to facilitate lymphoid differentiation or suppress malignant transformation for future clinical applications.
